# Comparative Effectiveness of SGLT2 Inhibitors and Semaglutide in Diabetic Nephropathy: A Retrospective Observational Study

**DOI:** 10.7759/cureus.87399

**Published:** 2025-07-06

**Authors:** Jimmy Joseph

**Affiliations:** 1 Internal Medicine, Aster DM Healthcare, Dubai, ARE

**Keywords:** diabetic nephropathy, egfr, glp-1 receptor agonist, semaglutide, sglt2 inhibitor, urine acr

## Abstract

Sodium-glucose cotransporter-2 (SGLT2) inhibitors and glucagon-like peptide-1 (GLP-1) receptor agonists have demonstrated renal benefits beyond glycemic control in patients with type 2 diabetes mellitus (T2DM). This study compares renal outcomes and metabolic parameters in 10 patients with early diabetic nephropathy, five treated with SGLT2 inhibitors (empagliflozin or dapagliflozin) and five with semaglutide, a type of GLP-1 receptor agonist. Over a six-month follow-up, both groups showed improvement in albuminuria and estimated glomerular filtration rate* (*eGFR) stabilization. However, semaglutide recipients had greater reductions in body weight and HbA1c, while SGLT2 inhibitor users exhibited more consistent declines in albuminuria. Both agents were well tolerated. This comparative series highlights the complementary nephroprotective profiles of both drug classes, supporting their individualized use in managing diabetic kidney disease. Further randomized trials are warranted to determine optimal sequencing or combination strategies.

## Introduction

Diabetic nephropathy remains a leading cause of end-stage kidney disease globally, affecting approximately 30-40% of patients with type 2 diabetes mellitus (T2DM) [1]. Traditionally managed with renin-angiotensin system (RAS) inhibitors and glycemic control, the emergence of newer antidiabetic agents such as sodium-glucose cotransporter-2 (SGLT2) inhibitors and glucagon-like peptide-1 (GLP-1) receptor agonists (RAs) has transformed the therapeutic landscape [2].

SGLT2 inhibitors (empagliflozin, dapagliflozin, canagliflozin) reduce glucose reabsorption in the proximal tubule, leading to natriuresis and glycosuria. Large trials like the EMPA-REG OUTCOME (BI 10773 (Empagliflozin) Cardiovascular Outcome Event Trial in Type 2 Diabetes Mellitus Patients) [3] and Dapagliflozin and Prevention of Adverse Outcomes in Chronic Kidney Disease (DAPA-CKD) [4] demonstrated significant slowing of kidney function decline, reduction in albuminuria, and cardiovascular protection. In the EMPA-KIDNEY (Study of Heart and Kidney Protection with Empagliflozin) trial, empagliflozin reduced the risk of kidney disease progression by 28% across a spectrum of chronic kidney disease (CKD) patients [5].

GLP-1 RAs (e.g., semaglutide, liraglutide) enhance glucose-dependent insulin secretion, suppress glucagon, and delay gastric emptying. Trials such as Liraglutide Effect and Action in Diabetes: Evaluation of Cardiovascular Outcome Results (LEADER) [6] and SUSTAIN-6 (Trial to Evaluate Cardiovascular and Other Long-term Outcomes With Semaglutide in Subjects With Type 2 Diabetes) [7] reported renal benefits, primarily reductions in albuminuria and cardiovascular events. Semaglutide has emerged as a potent agent offering weight reduction, improved glycemic control, and possible anti-inflammatory effects [8].

Mechanistically, SGLT2 inhibitors exert nephroprotection via modulation of tubuloglomerular feedback and decreased intraglomerular pressure, while GLP-1 RAs reduce oxidative stress, inflammation, and fibrosis [9,10]. Although both agents confer renal benefit, their comparative utility in real-world nephropathy management is not well established. This study explores the renal and metabolic outcomes of semaglutide vs. SGLT2 inhibitors in 10 patients with early diabetic nephropathy in a clinical setting. Our aim is to elucidate the practical differences in renal outcomes and guide clinicians in patient-centered therapy selection.

## Materials and methods

This was a retrospective observational study conducted at a single outpatient medical center, Aster DM Healthcare, Dubai, United Arab Emirates, evaluating the renal outcomes of semaglutide versus SGLT2 inhibitors in patients with T2DM and early-stage diabetic nephropathy.

In this observational case series, patients were divided into two treatment groups: a semaglutide group (n=5) and an SGLT2 inhibitor group (n=5), which included those receiving empagliflozin (n=3) and dapagliflozin (n=2). The selection of patients was based on clearly defined inclusion and exclusion criteria to ensure homogeneity and comparability.

Eligibility criteria

Eligible participants were adults aged 30-65 years with a confirmed diagnosis of T2DM for at least five years. All patients exhibited early-stage diabetic nephropathy, defined by an estimated glomerular filtration rate (eGFR) greater than 45 mL/minute/1.73m² (corresponding to CKD stages G2 to G3a) and a urine albumin-to-creatinine ratio (UACR) between 30 and 300 mg/g. Participants were required to be on a stable background therapy that included angiotensin-converting enzyme (ACE) inhibitors and angiotensin receptor blockers: RAS inhibitors, metformin, and statins, and to demonstrate a willingness to comply with follow-up visits for a duration of 12 months.

Patients were excluded if they had type 1 diabetes mellitus, advanced CKD (eGFR < 45 mL/min/1.73m²), non-diabetic kidney disease, or proteinuria from non-diabetic causes. Additional exclusion criteria included a recent cardiovascular event within the past six months, active liver disease with alanine transaminase (ALT) or aspartate aminotransferase (AST) levels exceeding three times the upper limit of normal, concurrent use of both a GLP-1 RA and an SGLT2 inhibitor, pregnancy or lactation, and documented non-adherence to therapy or missed follow-up appointments. These criteria were designed to minimize confounding factors and isolate the effects of each treatment modality on renal outcomes.

Follow-up and data collection

All patients were followed for 12 months, with periodic monitoring of renal parameters (eGFR, UACR), glycemic control (HbA1c), blood pressure, lipid profile, and body weight. Data were collected at baseline, six months, and 12 months.

Data analysis

All continuous variables were expressed as mean ± SD. Between-group comparisons were performed using independent two-sample t-tests (Welch's t-test, assuming unequal variances) for changes in HbA1c, weight, and eGFR. Due to the small sample size, statistical significance was considered at p < 0.05. Categorical variables, including adverse effects, were described narratively. Statistical analysis was performed using Python (SciPy library; https://scipy.org/).

## Results

Baseline characteristics, as detailed in Table 1, were comparable between the two groups. Parameters such as age, duration of diabetes, body mass index (BMI), prevalence of hypertension, and use of statins and RAS blockers were matched. Baseline renal function (eGFR) and albuminuria (UACR) were also similar, ensuring a balanced comparison of renal outcomes between the treatment arms.

**Table 1:** Baseline clinical characteristics of patients RAS: renin angiotensin system; eGFR: glomerular filtration rate; UACR: urine albumin creatinine ratio

Parameter	SGLT2 Group (n=5)	Semaglutide Group (n=5)
Age (years), mean ± SD	56.2 ± 4.1	53.4 ± 5.3
Gender (Male/Female), n	3/2	4/1
Duration of diabetes (years), mean ± SD	9.4 ± 2.3	8.8 ± 3.1
BMI (kg/m²), mean ± SD	31.1 ± 2.2	32.4 ± 3.0
Hypertension (%)	100%	100%
Statin use (%)	100%	100%
RAS blockade use (%)	100%	100%
Baseline eGFR (mL/minute), mean ± SD	68 ± 6	71 ± 7
Baseline UACR (mg/g), mean ± SD	420 ± 110	390 ± 95

At six months (Table 2), both groups showed improved glycemic control and renal parameters. Semaglutide led to greater reductions in HbA1c (−1.6% vs. −0.9%) and weight (−4.7 kg vs. −1.8 kg), while SGLT2 inhibitors achieved a larger decrease in albuminuria (−35% vs. −28%). Both groups experienced modest declines in eGFR and similar blood pressure reductions. These results highlight semaglutide’s metabolic benefits and the stronger albuminuria-lowering effect of SGLT2 inhibitors, supporting their complementary roles in managing diabetic nephropathy.

**Table 2:** Changes in clinical parameters at six months Unpaired t-tests were applied to continuous, normally distributed variables; Mann–Whitney U test was used for ΔUACR (%) due to percentage nature and small sample. p-values < 0.05 were considered statistically significant UACR: urine albumin creatinine ratio; HbA1C: glycosylated hemoglobin; eGFR: estimated glomerular filtration rate

Parameter	SGLT2 Group (n=5)	Semaglutide Group (n=5)	t-stat	p-value
Baseline HbA1c (%), mean ± SD	8.7 ± 0.6	8.9 ± 0.5	-	-
Δ HbA1c (%), mean ± SD	−0.9 ± 0.3	−1.6 ± 0.4	3.13	0.015
Δ Weight (kg), mean ± SD	−1.8 ± 0.9	−4.7 ± 1.2	4.32	0.003
Δ UACR (%)	−35%	−28%	–	0.04
Δ eGFR (ml/minute), mean ± SD	−1.5 ± 0.7	−2.2 ± 1.1	1.20	0.270
Blood pressure (mmHg)	−6.2 / −4.1	−5.8 / −3.9	–	–

At 12 months, both treatment groups showed sustained improvements (Table 3). Comparisons between groups used Welch's t-test. Semaglutide resulted in greater reductions in HbA1c (−2.1% vs −1.2%) and weight (−6.5 kg vs −2.5 kg), while SGLT2 inhibitors achieved a more pronounced reduction in albuminuria (−45% vs. −35%). eGFR declined modestly in both groups. Both drugs were well tolerated. One SGLT2 patient developed a mild urinary tract infection (UTI), and two semaglutide patients experienced nausea, with one discontinuation. These findings suggest durable metabolic and renal benefits from both therapies, with semaglutide excelling in glucose and weight control, and SGLT2 inhibitors offering superior albuminuria reduction. Individualized therapy is essential based on patient needs and tolerability.

**Table 3:** Changes in clinical parameters at 12 months Unpaired t-tests were applied to continuous, normally distributed variables; Mann–Whitney U test was used for ΔUACR (%) due to percentage nature and small sample. p-values < 0.05 were considered statistically significant UACR: urine albumin creatinine ratio; HbA1C: glycosylated hemoglobin; eGFR: estimated glomerular filtration rate

Parameter	SGLT2 Group (n=5)	Semaglutide Group (n=5)	t-stat	p-value
Baseline HbA1c (%), mean ± SD	8.7 ± 0.6	8.9 ± 0.5	t = 2.564	0.035
Baseline UACR (mg/g), mean ± SD	420 ± 110	390 ± 95	t = 1.618	0.148
Baseline eGFR (ml/minute), mean ± SD	68 ± 6	71 ± 7	t = -0.698	0.506
Δ HbA1c (12 months), mean ± SD	-1.2 ± 0.4	-2.1 ± 0.5	t = 2.026	0.077
Δ Weight (kg), mean ± SD	-2.5 ± 1.0	-6.5 ± 1.5	t = 4.706	0.002
Δ UACR (%)	-45%	-35%	-	0.04
Δ eGFR (ml/minute), mean ± SD	-2.8 ± 1.2	-4.0 ± 1.5	t = 1.595	0.150
Adverse Effects	1 mild UTI	Nausea in 2, 1 discontinued	—	—

Table 4 presents individual outcomes over six and 12 months in 10 patients with diabetic nephropathy treated with either SGLT2 inhibitors or semaglutide. Both groups demonstrated improvements in eGFR, UACR, HbA1c, and weight. SGLT2i patients showed a steady decline in albuminuria and slower eGFR reduction, with average UACR falling from 422 mg/g to 235 mg/g. Semaglutide led to greater reductions in HbA1c (up to 1.8%) and weight (up to 5.2 kg). eGFR decreased modestly in both groups. These findings indicate consistent renal and metabolic benefits from both therapies, supporting their use in early diabetic nephropathy with tailored patient selection.

**Table 4:** Individual patient outcomes at 6 and 12 months (10 patients) SGLT2i: sodium-glucose cotransporter 2 inhibitors

Patient ID	Group	Baseline eGFR (ml/minute)	6-month eGFR (ml/minute)	12-month eGFR (ml/minute)	Baseline UACR (mg/g)	6-month UACR (mg/g)	12-month UACR (mg/g)	HbA1c↓ (%)	Weight↓ (kg)
P1	SGLT2i	72	70.5	69	450	290	250	1.0	1.6
P2	Semaglutide	75	73.2	71	400	305	260	1.7	5.2
P3	SGLT2i	65	64	62.5	380	230	200	0.8	2.1
P4	Semaglutide	70	67.5	66	360	270	230	1.5	4.9
P5	SGLT2i	69	68.3	66.8	410	275	240	0.9	1.3
P6	Semaglutide	68	65.4	64	395	315	275	1.8	4.3
P7	SGLT2i	67	65.9	64	440	295	250	0.7	2.0
P8	Semaglutide	72	69.8	68	385	310	270	1.6	5.0
P9	SGLT2i	66	64.7	63	430	285	240	1.1	1.9
P10	Semaglutide	71	68.2	66.5	370	300	255	1.4	4.8

This 10-patient case series compared the effects of SGLT2 inhibitors and semaglutide in patients with T2DM and early diabetic nephropathy over 12 months. Baseline characteristics were similar across both groups. Semaglutide achieved greater reductions in HbA1c (−2.1% vs. −1.2%) and weight (−6.5 kg vs. −2.5 kg), highlighting its metabolic advantages. In contrast, SGLT2 inhibitors led to a more pronounced reduction in albuminuria (−45% vs. −35%) and slightly better preservation of eGFR. At 12 months, eGFR decline was modest in both groups. Adverse events were minimal: one mild UTI in the SGLT2 group and gastrointestinal symptoms in two semaglutide patients, with one discontinuation.

## Discussion

The study findings suggest that while both therapies confer renal and metabolic benefits, semaglutide may be more effective for glycemic and weight control, whereas SGLT2 inhibitors may be superior in albuminuria reduction. The therapies appear complementary, and selection should be guided by individual patient characteristics and clinical priorities. Semaglutide had more pronounced effects on glycemic control and body weight. No serious adverse events were reported. Two patients on semaglutide experienced gastrointestinal symptoms, while one patient required discontinuation.

The outcomes in our series are consistent with findings from large trials [3,4]. SGLT2 inhibitors demonstrated significant renoprotection, largely through reduction in glomerular hyperfiltration and improved hemodynamic regulation. This translated into a substantial decrease in albuminuria among our cohort, validating their mechanistic efficacy. Semaglutide, meanwhile, was superior in terms of glycemic control and weight loss, aligning with findings from SUSTAIN-6 [7] and PIONEER 6 (Peptide Innovation for Early Diabetes Treatment) [8] studies. The modest decline in albuminuria and eGFR stabilization reflects the growing recognition of GLP-1 RAs as renal protective agents, possibly through anti-inflammatory and anti-atherogenic effects [9,10]. Interestingly, both groups exhibited minor reductions in eGFR, which is expected in the early phase due to a reduction in intraglomerular pressure within the kidneys but ultimately stabilizes with long-term therapy [5].

The weight reduction and HbA1c drop with semaglutide may offer additive benefits in obese patients with metabolic syndrome. In real-world practice, patient factors such as cardiovascular status, weight concerns, and tolerance should guide therapeutic decisions. SGLT2 inhibitors are preferred for patients with overt proteinuria and cardiovascular risk, while GLP-1 RAs like semaglutide may benefit those patients needing weight loss or glucose control without volume depletion [11]. Combination therapy remains an emerging area. Evidence suggests that using both agents together can have additive or synergistic effects on renal and cardiovascular endpoints [12]. However, data is limited, and careful monitoring is warranted.

Limitations of our study include it being a single-center study, the small sample size, short duration, and lack of randomization. Nonetheless, the game uniformity in baseline characteristics and consistent follow-up enhances internal validity. The observed trends warrant further investigation in larger prospective cohorts or pragmatic trials.

## Conclusions

This study highlights the complementary nephroprotective effects of SGLT2 inhibitors and semaglutide in patients with early diabetic nephropathy. While SGLT2 inhibitors demonstrated superior albuminuria reduction, semaglutide achieved greater improvements in glycemic control and weight loss. Both agents effectively stabilized renal function over a six-month period, with minimal adverse effects. Our findings underscore the importance of individualized therapy in diabetic kidney disease.

SGLT2 inhibitors may be preferred in patients with significant albuminuria and cardiovascular risk, while semaglutide may be advantageous in obese or poorly controlled diabetic patients. Given the differing mechanisms of action, combination strategies could potentially yield superior outcomes but require more evidence. In conclusion, both SGLT2 inhibitors and semaglutide offer valuable tools in managing diabetic nephropathy. Clinicians should consider patient-specific factors, comorbidities, and treatment goals when selecting therapy. Further studies are warranted to optimize treatment algorithms and explore the long-term renal outcomes of combined use.
